# Fine mapping of a large-effect QTL conferring *Fusarium* crown rot resistance on the long arm of chromosome 3B in hexaploid wheat

**DOI:** 10.1186/s12864-015-2105-0

**Published:** 2015-10-23

**Authors:** Zhi Zheng, Jian Ma, Jiri Stiller, Qiang Zhao, Qi Feng, Frédéric Choulet, Catherine Feuillet, You-Liang Zheng, Yuming Wei, Bin Han, Guijun Yan, John M. Manners, Chunji Liu

**Affiliations:** CSIRO Agriculture, 306 Carmody Road, St Lucia, QLD 4067 Australia; School of Plant Biology, Faculty of Science and The UWA Institute of Agriculture, The University of Western Australia, Perth, WA 6009 Australia; National Foxtail Millet Improvement Centre, Institute of Millet Crops, Hebei Academy of Agricultural and Forestry Sciences, Shijiazhuang, China; Triticeae Research Institute, Sichuan Agricultural University, Wenjiang, Chengdu, 611130 China; National Center for Gene Research, Shanghai Institutes for Biological Sciences, Chinese Academy of Sciences, 500 Caobao Road, Shanghai, 200233 China; INRA-UBP Joint Research Unit 1095, Genetics, Diversity and Ecophysiology of Cereals Clermont-Ferrand, Clermont-Ferrand, F-63100 France

**Keywords:** Fusarium crown rot, Fine mapping, Hexaploid wheat, Co-segregating, SSR marker

## Abstract

**Background:**

Fusarium crown rot (FCR) is a major cereal disease in semi-arid areas worldwide. Of the various QTL reported, the one on chromosome arm 3BL (*Qcrs.cpi-3B*) has the largest effect that can be consistently detected in different genetic backgrounds. Nine sets of near isogenic lines (NILs) for this locus were made available in a previous study. To identify markers that could be reliably used in tagging the *Qcrs.cpi-3B* locus, a NIL-derived population consisting of 774 F_10_ lines were generated and exploited to assess markers selected from the existing linkage map and generated from sequences of the 3B pseudomolecule.

**Results:**

This is the first report on fine mapping a QTL conferring FCR resistance in wheat. By three rounds of linkage mapping using the NILs and the NIL-derived population, the *Qcrs.cpi-3B* locus was mapped to an interval of 0.7 cM covering a physical distance of about 1.5 Mb. Seven markers co-segregating with the locus were developed. This interval contains a total of 63 gene-coding sequences based on the 3B pseudomolecule, and six of them were known to encode disease resistance proteins. Several of the genes in this interval were among those responsive to FCR infection detected in an earlier study.

**Conclusions:**

The accurate localization of the *Qcrs.cpi-3B* locus and the development of the markers co-segregating with it should facilitate the incorporation of this large-effect QTL conferring FCR resistance into breeding programs as well as the cloning of the gene(s) underlying the QTL.

**Electronic supplementary material:**

The online version of this article (doi:10.1186/s12864-015-2105-0) contains supplementary material, which is available to authorized users.

## Background

Fusarium crown rot (FCR) is a chronic and serious disease of cereals. Field surveys showed that F. *pseudograminearum* is the most prevalent pathogen for FCR in Queensland and New South Wales in Australia but many different species of *Fusarium* can cause this disease [1]. Due most likely to the high intensity of cereal in cropping system combined with wider adoption of minimum tillage for moisture conservation, FCR has become more prevalent in many parts of the semiarid regions in recent years [2]. A survey in 2009 found that FCR causes an estimated annual yield loss of $80 million Australia dollars in the wheat industry alone [3]. A study in the Pacific Northwest in USA showed that FCR could significantly reduce yield of both wheat and barley [4]. Additionally, FCR infected plants could also produce mycotoxins in grains as well as other tissues. The presence of these compounds in food and feeds can be harmful for human and livestock [5].

Growing resistant varieties has long been recognised as an integral part in effectively managing FCR [6]. Working toward the breeding of varieties resistant to this disease, sources of resistance were identified by germplasm screenings [6, 7]. Over the last decade, significant effort has also been made in identifying QTL conferring FCR resistance. Several QTL have been detected from several different sources of resistance [8]. Of the QTL reported so far, the one on 3BL consistently gave the largest effects and was identified from several genotypes [9–11]. Limited data indicates that the 3BL locus also confers field resistance to FCR and it reduces whitehead incidence significantly under field conditions [8].

QTL mapping has become a routine procedure in locating genes controlling quantitative traits to specific genomic regions. However, QTL mapping has only limited resolution and markers obtained from such studies may not be reliably used in tagging the targeted loci [12]. The main reason why QTL mapping provides only limited resolution is due to the heterogeneity in genetic backgrounds in QTL mapping populations in regard to a targeted locus. Some non-targeted traits segregating in the QTL mapping populations could interfere with the accurate phenotyping of a targeted trait thus lines containing the same allele may exhibit different levels of resistance. Recent studies showed that both plant height and heading date have significant effects on FCR severity in both wheat and barley. LOD values and magnitudes of QTL conferring FCR resistance were both reduced when the effect of height and heading date were accounted for by the covariance analysis [13–15]. Thus these traits need to be fixed in segregating populations which are suitable for developing markers tightly linked to a FCR locus.

Fixing genetic backgrounds in regarding to a targeted locus can be achieved with near-isogenic lines (NILs) [16–19] or populations generated from NILs [20]. Taken advantage of the newly released chromosome 3B pseudomolecule [21], we used both NILs [22] and a NIL-derived population in developing tightly linked markers to the large-effect FCR locus on 3BL and results obtained are reported in this paper.

## Results

**Fig. 1:**
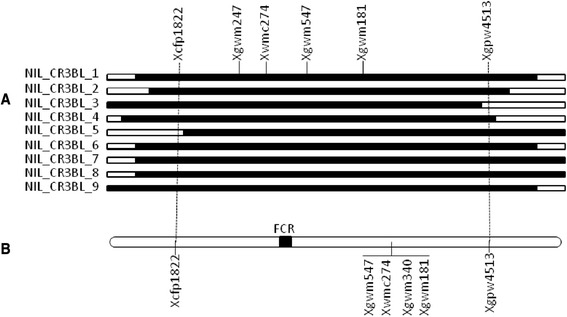
Map location of the FCR locus *Qcrs.cpi-3B* based on data from the nine sets of near-isogenic lines (**a**) and based on the use of 160 NIL-derived lines (**b**)

Twenty SSR markers selected from the existing linkage map [23] for the *Qcrs.cpi-3B* interval were assessed. Nine of these markers detected polymorphism between isolines of the nine sets of NILs used in this study. Five of these markers (*Xgwm247, Xwmc274, Xgwm547, Xgwm340* and *Xgwm181*) detected difference between the isolines for each of the nine NIL sets. Markers *Xcfp1822* detected difference between isolines for eight of the nine NIL sets and *Xgpw4513* detected differences between the isolines for seven of the nine NIL sets (Fig. 1a). Based on their order in the linkage map of chromosome 3B [23], *Xcfp1822* and *Xgpw4513* were tentatively selected as candidates flanking the *Qcrs.cpi-3B* locus.

**Fig. 2:**
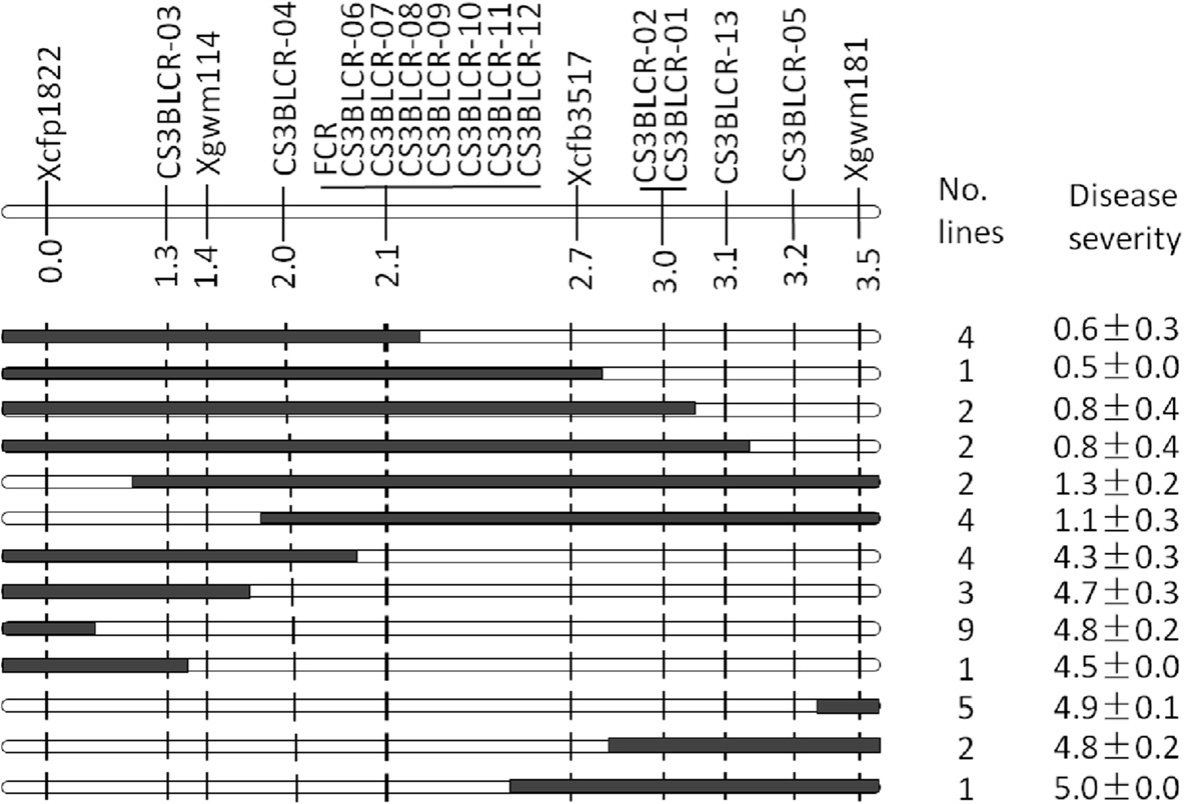
Genotypes and phenotypes of the haplotypes identified based on markers surrounding the FCR locus *Qcrs.cpi-3B*

To determine the order of these SSR markers and confirm the results obtained from the NILs, a subpopulation containing the first 160 lines of the fine mapping population were then analysed. Disease ratings of this subpopulation fell into two classes, one ranging from 0 to 1 (resistant) and the other from 4 to 5 (susceptible). There were no intermediate rantings and all individuals could be placed in one of the two classes (Additional file 1: Figure S1). The locus *Qcrs.cpi-3B* was successfully placed within the interval flanked by *Xcfp1822* and *Xgwm181* (Fig. 1b). Assessing the whole fine mapping population of 774 lines with these two flanking markers identified 40 recombinants. Aligning the two flanking markers against the 3B pseudomolecule (https://urgi.versailles.inra.fr/.) placed the distal marker *Xgwm181* at the position of 773.7Mbp and the proximal marker *Xcfp1822* at the position of 160Mbp. Eighteen new markers were designed based on the 3B pseudomolecule targeting this interval. Three of them (*CS3BLCR-01*, *CS3BLCR-02* and *CS3BLCR-03*) were polymorphic and they were all successfully mapped in the targeted interval (Table 1). The targeted locus was mapped between marker *CS3BLCR-01* and *CS3BLCR-03* and the physical locations of these two flanking markers on the 3B pseudomolecule were at the positions 773.0 Mbp and 750.8 Mbp, respectively. Against this newly defined interval, another 20 markers were then designed based on the 3B pseudomolecule at a distance of about 10 Mbp between two adjacent markers. Four of these markers (*Xgwm114*, *CS3BLCR-04*, *Xcfb3157* and *CS3BLCR-05*) detected polymorphism in the fine mapping population (Table 1) and their placements on the linkage map reduced the FCR locus to a 0.7 cM or 1.5Mbp interval flanked by *CS3BLCR-04* and *Xcfb3517* (Fig. 2).

**Table 1:** Primers for new markers successfully mapped near the *Qcrs.cpi-3B* locus#

Marker	Forward primer/position (bp)	Reverse primer/position (bp)	Product size (bp)
CS3BLCR-01	AAACAGAGTAACAGACCGACC/773070474	TTCAGATAAGGAAACCAAACA/773070830	357
CS3BLCR-02	CATACATACTCCCTCTGTTACCT/773095727	CTTCCCTTTGCTGCGTGT/773096117	391
CS3BLCR-03	GGTCTCTTCGCTGACAAA/750842960	CCTTGCTTGCTTATGTGCT/750843318	359
CS3BLCR-04	GCTTTGAGTTATGTATCCGTG/770066939	TAAGAAACCAGCGTCAGAAA/770067148	210
CS3BLCR-05	ATCTGGGATAATGAGGTGG/770736229	GAAGACCAACATAGTAATCGG/770736554	326
CS3BLCR-06	TCCTTATTCCTCTTCCCTAAA/770210969	TTGACTTGTTTCTTATGTCCTG/770211217	249
CS3BLCR-07	GAGGGAGGTCTGGTTGGG/770225517	AAGCGGAGTGTTGTGTTG/770225671	155
CS3BLCR-08	TGATTACAACAAACAAACCC/770294691	ATACACTGAACAAACGGATAA/770294978	288
CS3BLCR-09	GTAGAAATAGGGACAACGGAG/770304690	ACAAGAAACGGCAGATGA/770305044	355
CS3BLCR-10	AAAGCCACCTAATGAAGAAAC/770331178	GAAGAAGAAGAGGAGCGG/770331485	308
CS3BLCR-11	TGTTGTTTGGGTAGTCATTCT/770349503	GCTCCAGTGCTTGTAGTCA/770349800	298
CS3BLCR-12	AAGAAGAGGAGCAGCAAAG/770552012	AAAGCCACCTAATGAAGAAAC/770552259	248
CS3BLCR-13	ATGGAGGAGACGAAACTCA/770843419	GTTATCTTGGTATGCGGGT/770843678	260

#Primer positions and the product sizes were based on those reported in the chromosome 3B pseudomolecule; the annealing temperature used for all of the primer sets was 55 °C

To further define the FCR locus, the 3B pseudomolecule was used again to develop new markers for the targeted interval. Sixty markers were designed in this third round of marker development with an average density of about 0.2 Mbp between the markers (Additional file 2: Table S1). Seven of the 60 markers (including *CS3BLCR-06, CS3BLCR-07, CS3BLCR-08. CS3BLCR-09, CS3BLCR-10, CS3BLCR-11* and *CS3BLCR-12)* detected polymorphism in the mapping population and they all co-segregated with the FCR locus (Fig. 2).

Based on the 3B pseudomolecule, there were 63 CDSs in this 0.7 cM interval (or 1.5 Mb) containing the FCR locus. Six of them were known to encode disease resistance proteins (Additional file 3: Table S2). Further, we aligned RNA reads obtained from a recent study [24] and retrieved original paired reads for all aligned reads and de novo assembled them using CLC Genomic Workbench 7. This de novo assembly of the expressed reads identified 121 assembled contigs in the targeted interval. Of them, five were related to disease resistance (Additional file 4: Table S3). This targeted interval also contains six of the differentially expressed genes following FCR infection and genes with SNPs between the resistant and susceptible NILs [24]. Together, the 17 genes and contigs from these three sources represent 15 unique sequences. Of them, nine were known to encode disease resistance proteins (Table 2).

**Table 2:** Unique sequences in the targeted interval harbouring the *Qcrs.cpi-3B* locus

Gene/sequences	Putative function
TRAES3BF024600100CFD_c1	Disease resistance-responsive family protein
TRAES3BF279000170CFD_c1	Disease resistance rpp13-like protein 3-like
TRAES3BF279000190CFD_c1	Disease resistance protein rga2
TRAES3BF279000200CFD_c1	Disease resistance rpp13-like protein 3-like
TRAES3BF279000210CFD_c1	Disease resistance rpp13-like protein 2-like
TRAES3BF279000290CFD_c1	NBS-LRR resistance partial
contig_11	Disease resistance protein rpm1
contig_55	Disease resistance protein rga4
contig_118	Disease resistance protein rga4
Ta#S32545039^a^	AP3-complex subunit beta-A-like
Ta#S18011610^a^	Metallothionein
Ta#S26024264^a^	Unknown
Ta#S16266522^a^	Unknown
Ta#S37789723^a^	Unknown
Ta#S32500938^a^	Gibberellin 2-beta-dioxygenase 8-like

^a^UniGenes from the National Center for Biotechnology Information (NCBI) (downloaded from ftp://ftp.ncbi.nih.gov/repository/UniGene/Triticum_aestivum/)

## Discussion and conclusions

FCR is a serious threat to cereal production in semi-arid areas worldwide and growing resistant varieties is an essential component in effectively managing the disease. Working toward the breeding of resistant varieties, sources of resistance have been identified and several QTL controlling FCR have been reported. In the study reported here, we attempted to develop markers that can be reliably used to tag the FCR locus on 3BL. Using markers developed from the chromosome 3B pseudomolecule, the large-effect QTL was successfully mapped to an interval of 0.7 cM or 1.5 Mbp based on the nine sets of NILs and a NIL-derived population consisting of 774 lines. Within this interval, seven markers co-segregating with the targeted locus were developed and 15 unique genes or contigs related to disease resistance were identified. These results should facilitate not only the incorporation of this locus into breeding programs but also the effort of cloning gene(s) underlying the FCR locus.

The 18 markers mapped in this study covers a distance of 3.5 cM (Fig. 1). These markers covered a distance of 7.1 cM in the linkage map reported previously [23]. It is known that recombination rates at meiosis can be affected by many factors. In addition to the relateness of the parental genotypes [25], recombination frequencies can be regulated by major genes [26] and may also differ between egg and pollen mother cells [27, 28]. It is also well known that the presence of structural rearrangements between the parental genotypes can significantly reduce the recombination rates [29]. As the NILs and the NIL-derived population used for fine mapping in this study were all obtained from crosses between bread wheat (*Triticum aestivum* L.) and *T. spelta* genotypes [9, 22], the relatively distant genetic relatedness between the parental genotypes likely contributed to the reduced linkage distances obtained in this study. A population significantly larger than the one used in this study is being produced and will be used to further define the targeted interval and identify the gene(s) conferring FCR resistance at this locus.

The availability of the chromosome 3B pseudomolecule [21] significantly facilitated the study reported here and there is no reason why the same approach can not be used to fine map genes of other traits on this chromosome. However, of the 98 pairs of primers designed in this study only 13 detected polymorphism and were successfully mapped in the targeted interval. This rate of success does not seem to be very high but could be even lower if populations generated from more closely related genotypes were used. The 3B pseudomolecule makes it feasible now to exploit a high-throughput genotyping approach in identifying genes controlling any trait on this chromosome. The one based on low-coverage whole genome resequencing [30] is very appealing especially in considering that chromosome 3B is readily sortable by flow cytometry [31].

## Methods

### Plant materials

Nine sets of NILs for the 3BL FCR locus derived from three segregating populations (including 120 F_4_ lines from ‘Aus13832’/’CSCS6’, 108 BC_1_F_4_ lines from ‘Janz’*2/’CSCR6’ and 125 F_7_ lines from ‘Lang’/’CSCR6’) were available [22] and they were used in identifying markers flanking the locus in this study. The resistance donor ‘CSCR6’ belongs to the taxon *T. spelta*. It is one of the genotypes which showed the best resistance among the over 2200 genotypes screened [7]. The three susceptible parents are all Australian varieties. Detailed mapping of the 3BL locus was conducted using a NIL-derived population consisting of 774 F_10_ lines. This fine mapping population was developed as part of this study from a single plant of Janz/CSCR6 [9] based on the heterogeneous inbred family (HIF) method [32]. An SSR marker, *gwm181* locating near the peak of the FCR QTL on 3BL [9], was used to identify heterozygous lines in developing NILs. A single hetetozygous plant was used in developing the fine mapping population (at F_10_ generation) using the fast-generation technique [33].

### Genotyping and linkage analyses

Leaf tissues from the NILs and the fine mapping population were collected and stored at −80 °C until processing. Genomic DNA was extracted using the CTAB protocol [34]. PCR reactions for the marker analyses were carried out using α [^33^P] dCTP (3000 ci/mmol) following the manufacturer’s protocol (Multiplex-Ready Marker User Handbook, version 2.0). The amplified products were mixed with an equal volume of loading dye, denatured at 95 °C for 10 min, and 3.8 μl amplified samples were separated on a 5 % polyacrylamide gel containing 8 M urea at 100 W for 2 h. The gels were subsequently dried using a gel dryer for 50 min at 80 °C and exposed to Kodak X-Omat X-ray film for 4–6 days.

To construct a linkage map for the 3BL region, SSR markers from the existing linkage maps [23, 35] and new PCR-based markers developed based on the sequence of the 3B pseudomolecule [21] were screened for polymorphism against the nine sets of NILs [22]. Gene-coding sequences (CDSs) and 3B pseudomolecule sequence were downloaded from https://urgi.versailles.inra.fr/gb2/gbrowse/wheat_annot_3B/. Primers for all of the new markers were designed with SSRPrimerII [36]. Linkage analysis was carried out using the computer package JoinMap 4.0 [37].

### Evaluation of resistance to FCR

A highly aggressive *F. pseudograminearum* isolate, CS3096, was used in FCR assessment. This isolate was collected in northern New South Wales, Australia and maintained in CSIRO collection [1]. The methods used for inoculums preparation, inoculation and FCR assessment were based on that described by Li et al*.* [38]. Briefly, inoculums was prepared using plates of ½ strength potato dextrose agar (PDA). The inoculated plates were incubated for 7 days at room temperature before the mycelium was scraped. The plates were then incubated for a further 5–7 days under a combination of cool white and black (UVA) fluorescent lights with a 12 h photoperiod. The spores were harvested and the concentration of spore suspension was adjusted to 1 × 10^6^ spores per millilitre in distilled water. Tween 20 was added (0.1 % v/v) to the spore suspension prior to use for inoculation.

Seeds were germinated in Petri dishes on two layers of filter paper saturated with water. The germinated seedlings were immersed in the spore suspension for 1 min and two seedlings were planted into square punnets of a 56-well tray (Rite Grow Kwik Pots, Garden City Plastics, Australia) containing stem sterilized University of California mix C (50 % sand and 50 % peat v/v). The punnets were arranged in a randomized block design in a controlled environment facility (CEF). Settings for the CEF were: 25/16 (±1) °C day/night temperature and 65/85(±5)% day/night relative humidity, and a 14-hour photoperiod with 500 *μ*mol m^−2^s^−1^ photon flux density at the level of the plant canopy. To promote FCR development, water-stress was applied during the FCR assessment. Inoculated seedlings were watered only when wilt symptoms appeared. Three trials were conducted using the fine mapping population and five additional trials were then carried out to assess those recombinant lines identified based on markers flanking the targeted interval containing the FCR locus. Each trial contains two replicates, each with 14 seedlings. FCR severity was assessed 35 days post inoculation, using a 0 (no obvious symptom) to 5 (whole plant severely to completely nectrotic) scale as described by Li et al. [38].

### Identification of genes of interest in the target region

Based on the fine mapping results described above, CDSs located in the targeted genomic region were retrieved from the 3B pseudomolecule [21]. Paired RNA reads from the first of the nine sets of NILs obtained from an earlier study [24] were re-analysed for identifying transcripts of interest in the targeted genomic region. RNA datasets were trimmed by SolexaQA scripts (http://solexaqa.sourceforge.net/) to a minimum quality value of 30 and a minimum length of 70. Alignments of RNA reads from resistant and sensitive NILs were performed using the Biokanga suite (a tool developed at CSIRO, not published) with 2 mismatches allowed per read. Paired reads for all reads aligned to the defined region containing the FCR locus were retrieved by in house built Perl scripts and de novo assembled using CLC Genomics Workbench 7.

### Ethical standard

The authors state that the experiments conducted in this study comply with the current laws in Australia where they were conducted.

### Availability of data and materials

All the supporting data are included as supplementary files.

## Additional files

Additional file 1: Figure S1. Distribution of FCR severity in the subpopulation containing 160 lines. Disease severity was scored using a scale of 0 (no obvious symptom) to 5 (whole plant severely to completely nectrotic). (DOCX 39 kb) Additional file 2: Table S1. Markers not polymorphic in the interval harboring the Fusarium crown rot resistance locus *Qcrs.cpi-3B. (DOCX 26 kb)*
Additional file 3: Table S2. Annotations of the 63 coding sequences identified between markers *CS3BLCR-04* and *Xcfb3517* from the 3B pseudomolecule of Chinese Spring^*#*^. (DOCX 25 kb) Additional file 4: Table S3. Annotations of assembled contigs from expressed RNA reads locating in the targeted interval harboring the FCR resistance locus *Qcrs-cpi-3B* ^#^. (DOCX 30 kb)
