# Supplementary material for: Fine mapping of a large-effect QTL conferring Fusarium crown rot resistance on the long arm of chromosome 3B in hexaploid wheat
Source: BMC Genomics. 2015 Oct 23;16:850. doi: 10.1186/s12864-015-2105-0 (PMC4618961; doi:10.1186/s12864-015-2105-0)
Supplement: Additional file 1: Figure S1. — Distribution of FCR severity in the subpopulation containing 160 lines. Disease severity was scored using a scale of 0 (no obvious symptom) to 5 (whole plant severely to completely nectrotic). (DOCX 39 kb) [file 12864_2015_2105_MOESM1_ESM.docx]

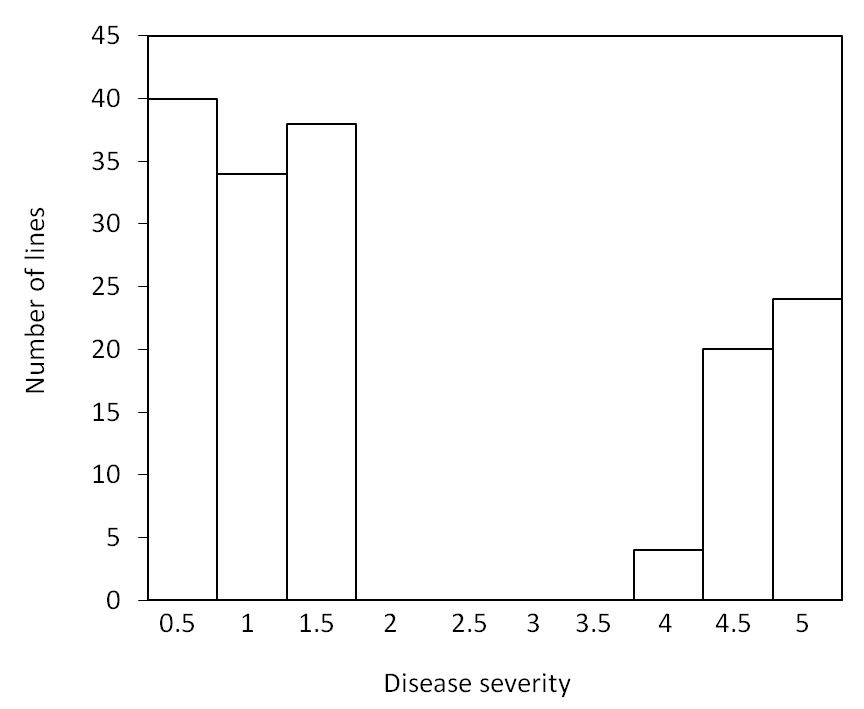


Supplementary Fig.S1 Distribution of FCR severity in the subpopulation containing 160 lines. Disease severity was scored using a scale of 0 (no obvious symptom) to 5 (whole plant severely to completely nectrotic).
