# Supplementary material for: Fine mapping of a large-effect QTL conferring Fusarium crown rot resistance on the long arm of chromosome 3B in hexaploid wheat
Source: BMC Genomics. 2015 Oct 23;16:850. doi: 10.1186/s12864-015-2105-0 (PMC4618961; doi:10.1186/s12864-015-2105-0)
Supplement: Additional file 2: Table S1. — Markers not polymorphic in the interval harboring the Fusarium crown rot resistance locus Qcrs.cpi-3B. (DOCX 26 kb) [file 12864_2015_2105_MOESM2_ESM.docx]

Table S1: Markers not polymorphic in the interval harboring the Fusarium crown rot resistance locus *Qcrs.cpi-3B*
