# Supplementary material for: Fine mapping of a large-effect QTL conferring Fusarium crown rot resistance on the long arm of chromosome 3B in hexaploid wheat
Source: BMC Genomics. 2015 Oct 23;16:850. doi: 10.1186/s12864-015-2105-0 (PMC4618961; doi:10.1186/s12864-015-2105-0)
Supplement: Additional file 3: Table S2. — Annotations of the 63 coding sequences identified between markers CS3BLCR-04 and Xcfb3517 from the 3B pseudomolecule of Chinese Spring#. (DOCX 25 kb) [file 12864_2015_2105_MOESM3_ESM.docx]

Table S2. Annotations of the 63 coding sequences identified between markers *CS3BLCR-04* and *Xcfb3517* from the 3B pseudomolecule of Chinese Spring*^#^*

^#^NA indicates that no hits were detected.
