# Supplementary material for: Fine mapping of a large-effect QTL conferring Fusarium crown rot resistance on the long arm of chromosome 3B in hexaploid wheat
Source: BMC Genomics. 2015 Oct 23;16:850. doi: 10.1186/s12864-015-2105-0 (PMC4618961; doi:10.1186/s12864-015-2105-0)
Supplement: Additional file 4: Table S3. — Annotations of assembled contigs from expressed RNA reads locating in the targeted interval harboring the FCR resistance locus Qcrs-cpi-3B #. (DOCX 30 kb) [file 12864_2015_2105_MOESM4_ESM.docx]

Table S3. Annotations of assembled contigs from expressed RNA reads locating in the targeted interval harboring the FCR resistance locus *Qcrs-cpi-3B*^#^


^#^NA indicates that no hits were detected.
